# Implementation of falls risk evaluation at one-year after total hip arthroplasty: a cross-sectional study

**DOI:** 10.1186/s40945-022-00141-6

**Published:** 2022-07-15

**Authors:** Tony Adebero, Pavlos Bobos, Lyndsay Somerville, James Howard, Edward M. Vasarhelyi, Brent Lanting, Susan W. Hunter

**Affiliations:** 1 Faculty of Health Sciences, Department of Health and Rehabilitation Sciences, University of Western Ontario, London, ON Canada; 2 Applied Health Research Centre, Li Ka Shing Knowledge Institute of St. Michael’s Hospital, Toronto, ON Canada; 3 Dalla Lana School of Public Health, Institute of Health Policy, Management and Evaluation, Department of Clinical Epidemiology and Health Care Research, University of Toronto, Toronto, ON Canada; 4 Schulich School of Medicine & Dentistry, Department of Surgery, University of Western Ontario, London, ON Canada; 5 School of Physical Therapy, University of Western Ontario, London, ON Canada

**Keywords:** Osteoarthritis, Accidental falls, Hip arthroplasty, Aged

## Abstract

**Background:**

Research has demonstrated an increased risk of falls after total hip arthroplasty (THA). Yet, people’s knowledge on falls risk factors and how falls prevention strategies are being used after THA have not been examined. If a person’s knowledge of falls and self-efficacy about falls prevention strategies is low this would indicate a pressing need for interventions to lessen risk. The study objectives were: 1) to determine the falls knowledge and what fall prevention strategies people used after (THA) and 2) to determine the outcomes of a falls risk assessment at 12-months after unilateral THA.

**Methods:**

Overall, 108 people completed the Falls Risk for Older People – Community Setting (FROP-Com) scale, a falls questionnaire (covered occurrence of falls, knowledge on falls risk factors, falls prevention strategies implemented after THA surgery), 6-m Walk Test (6mWT), 30-Second Chair Stand Test (30CST), Timed-up and Go (TUG) Test, and Activities-specific Balance Confidence Scale (ABC).

**Results:**

Twenty-five (23.2%) people fell at least once in the 12 months after THA. Scores on the FROP-Com ranged from 2–20 with an average of 8.2 ± 3.6 indicating a mild falls risk. The importance of falling compared to other health concerns was rated as moderate to high (6.8 ± 2.9) and the majority of participants (*n* = 98, 90.7%) believed falls can be prevented after THA. Total scores on the ABC scale ranged from 30.6% to 100.0% with an average score of 84.4 ± 15.5%, indicating high function. Only 47 people (43.5%) reported receiving falls prevention education. A total of 101 falls prevention strategies were completed by 67 people (62%), the most common strategy was environmental modifications (e.g., installation of grab bars) at 37.4%, while exercise was mentioned by only 2%. The majority of people had functional deficits in 30CST (62%) and TUG (76.9%) at 12-months after unilateral THA.

**Conclusions:**

Almost a quarter of the sample had experienced a fall in the 12-months after THA and functional deficits were common. The majority of the sample had proactively implemented falls prevention strategies after the surgery. Yet importantly, people after THA had limited exposure to falls prevention education and implemented a limited range of prevention strategies.

**Supplementary Information:**

The online version contains supplementary material available at 10.1186/s40945-022-00141-6.



## Background

Symptomatic hip osteoarthritis (OA) is a risk factor for falls in community-dwelling older adults [1]. The annual occurrence of falls in older adults with hip OA has been reported at 45% [2]. In the 12 months leading up to total hip or total knee joint replacement surgery, 41% of people sustained one or more falls and joint symptoms accounted for 35% of the falls [3]. The high risk of falls associated with OA has been attributed to pain, lower extremity muscle weakness, and gait and balance impairments [2, 3]. Surgical management of hip OA with elective total hip arthroplasty (THA) is very successful with at least 90% of people reporting satisfaction with the surgery [4].

Despite rehabilitation after THA, deficits in muscle strength, balance and walking are found even years after the operation [5–8]. These deficits may be the consequences of the OA that necessitated the surgical intervention and new deficits that are related to the THA surgery [8–10]. The only prospective study evaluating falls in the first year after only THA was done in older women, 30% of the sample fell and this was an increased risk compared to healthy community-dwelling controls [11]. Nagai et al. [12] also found a high prevalence of a fear of falling for performing daily activities in older women after THA. A fear of falling can also lead to adverse consequences in older adults such as reduced activity participation and a decreased quality of life [13].

The increased risk of falls after THA supports the need to evaluate falls prevention strategies that are currently in place that target this patient population [14]. No literature exists on the knowledge of falls risk factors and falls prevention strategies used among people after THA. Even for community-dwelling older adults without a THA there are gaps in their understanding of risks [19]. If a person’s knowledge and self-efficacy about falls and falls prevention strategies is low, then it is believed education that targets these gaps during the post-surgical period can reduce the risk [15]. Identification of deficits in knowledge and risk reduction strategies highlights an avenue for falls prevention interventions. For community-dwelling older adults, recommended falls risk screening guidelines for clinical practice include a multifactorial falls risk assessment (i.e., focused falls history, physical examination, functional and environmental assessments) of known falls risk factors [16]. While no guidelines have been developed specifically for people after THA, the application of existing guidelines to understand the risk profiles from a falls risk screening process in this population has not been published.

Falls risk evaluation in clinical practice as recommended by the most prominent falls prevention guidelines is a screening process performed at one point in time [16]. While Brander et al. [17] proposed falls risk assessment should be performed starting at 3–6 months after THA, there is no consensus that falls risk screening is an established evidence-informed practice within care pathways after THA. Physiotherapy has an important role to play in the prevention of falls in this patient population through our involvement in the provision of routine post-surgical rehabilitation.

Additionally, to identify the falls prevention requirements that are unique to this population it is important to identify existing falls knowledge and describe the strategies that people with THA implemented to reduce their risk of falls. In conjunction with educational needs, it is important to be able to quantify the presence of modifiable falls risk factors amenable to physiotherapy intervention to target known deficits seen after this type of surgery.

Therefore, the objectives of the study were: 1) to determine the falls knowledge and falls prevention strategies people used after THA surgery and 2) determine the outcomes of a falls risk assessment at 12-months after unilateral THA. We hypothesized that knowledge of falls would be low and physical measures of functional mobility, lower extremity strength and gait would be reduced compared to normative values for community-dwelling older adults indicating an increased falls risk in the future 12 months for people after a THA.

## Methods

### Study design

A cross-sectional study was designed for people attending their 1-year assessment after a unilateral THA. Data were collected between February and December 2017. This study was approved by the University of Western Ontario and the Clinical Resources Impact Committee of Lawson Health Research Institute in London, Ontario, Canada. Participants provided written informed consent.

### Subjects

Eligibility criteria were: 60 years of age and older, primary elective unilateral THA surgery for OA of the hip in the previous 12 months, and able to walk at least 10 m without the assistance of another person (but allowed a gait aid). Exclusion criteria were: non-ambulatory and attending a one-year follow-up for a revision THA or had surgery for a diagnosis other than OA.

### Material

#### Falls questionnaire

The questionnaire included 20 questions divided into 3 sections: i) falls since the THA surgery (6 questions), ii) knowledge of falls risk factors (13 questions), and iii) falls prevention strategies (1 question). A fall was defined as “an unexpected event in which the participant comes to rest on the ground, floor or a lower level” [18]. Injuries were defined as major or minor—a major injury was any fall when medical attention was required for care of a fracture, while a minor injury was a bruise or laceration in which medical attention may or may not have been required. Participants were asked if they remembered being taught how to prevent falls during their post-surgery physiotherapy rehabilitation. The importance of falling compared to their other health concerns was evaluated on a scale from 0 (not important at all) to 10 (most important).

The knowledge of falls risk factors section asked participants to rate on a scale from 0 (not likely) to 10 (most likely) how likely 13 risk factors would make an older adult fall [19]. Four themes of risk factors were covered – interior environmental factors (rugs, grab bars), exterior environmental factors (sidewalks and streets for ice/snow and maintenance), physical factors (balance, leg strength, bone health, vision, medications), and judgement factors (risky behaviours, lack of attention, forgetful). An open-ended question collected falls prevention strategies the participants had implemented since their THA surgery. The responses were categorized using the work of Hill et al. [20] into one of the following seven possible categories: none, behavioural, support while mobilizing (use of supportive equipment or items), approach to movement (moving in a particular manner), physical environment (modifications of their physical home environment), medical (strategies that influenced medical conditions), and activity and exercise.

#### Balance confidence

The Activities-specific Balance Confidence (ABC) Scale is a 16-item scale that evaluates self-efficacy in mobility-related tasks [21]. Each item is rated on a scale of 0% (no confidence) to 100% (completely confident) and the total score is an average of the 16 items. ABC scores less than 50 indicate a low level of functioning, scores between 50 and 80 are considered a moderate level of functioning, and scores above 80 represent a high level of functioning [22].

#### Physical performance tests

Three tests of physical performance evaluated gait, leg strength, and functional mobility. Time to complete the 6-m Walk Test was recorded with a stopwatch to the nearest hundredth of a second. The 30-Second Chair Stand Test (30CST) evaluated lower extremity strength as the number of stands completed in 30 s using a chair with a seat height of 45 cm [23]. The Timed-Up-and-Go Test (TUG) assessed a person’s functional mobility as the time, recorded to the nearest hundredth of a second using a stopwatch, to complete this activity [24].

#### Falls risk assessment

The Falls Risk for Older People – Community Setting (FROP-Com) questionnaire is a multi-factorial falls risk assessment tool that assesses 25 risk factors for falls [25]. The assessment consists of 28 self-report items scored on either a dichotomous scale of yes or no questions or an ordinal scale of 0 to 3. Individual responses are summed to generate a total score with a maximum score of 60, while scores of 0 to 10 are considered a mild falls risk, scores of 11 to 18 are a moderate risk and scores greater than 19 are a high falls risk that warrants further action [25].

### Procedure

The following demographic and clinical information were obtained: age, sex, surgical approach, number of medications, comorbidities, mobility aid use, physical activity level and history of a previous lower extremity joint arthroplasty. Physical activity levels were categorized within 3 groups: sedentary (somewhat inactive), moderately active, and vigorously active. Participants completed the Montreal Cognitive Assessment [26], Western Ontario and McMaster Universities Arthritis Index (WOMAC) [27], and the Short-Form 12 (SF-12) [28]. Surgeons completed the Harris Hip Score for each participant [29]. Participants then completed the falls questionnaire, three physical performance tests targeting areas adversely impacted after THA and a falls risk evaluation.

### Statistical analysis

Participant demographics were summarized using means and standard deviations or frequencies and percentages, as appropriate. Data from the falls questionnaire provided frequencies, continuous variables, and open-ended responses. The open-ended responses were categorized into the seven areas and presented as frequencies and percentages. The ABC score was summarized as mean and standard deviation. The average scores and percentage of participants with scores below normative values for the 30CST, TUG and gait velocity were determined. A gait velocity below 1.0 m/s is associated with increased adverse events in older adults [30] and was used as the threshold for evaluation in this study. Normative data for community-dwelling older adults used 30CST data by Rikli and Jones [31], and TUG and gait velocity data by Steffen et al. [32] Average scores and standard deviations of the FROP-Com were calculated.

## Results

### Demographics

There were 305 individuals screened for the study and 108 met the inclusion criteria and agreed to participate. The average age for the sample was 72.4 ± 6.5 years and 39.8% were males. (Table 1) In the 12 months after the surgery 25 (23.2%) people sustained 30 falls—5 (16.1%) occurred between hospital discharge and 3 months, 7 (22.6%) between 3 and 6 months, and 18 (57.1%) occurred between 6 and 12 months. Fourteen (13.0%) people sustained an injury, minor for 11 (78.6%) and major for 3 (21.4%). Ten people reported their use of a mobility aid was because of the THA.

**Table 1:** Demographics for people assessed at 12 months after a unilateral total hip arthroplasty for osteoarthritis. (*n* = 108)

Participant Characteristics	Mean ± SD [range] or Frequency (%)
Age (years)	72.4 ± 6.5 [60–88]
Sex (% female)	65 (60.2%)
Number of prescription medications	3.6 ± 3.4 [0–17]
Comorbidities	
Hypertension	53 (49.1%)
Diabetes mellitus	16 (14.8%)
Dyslipidemia	40 (37.0%)
Smoker	2 (1.9%)
Myocardial infarction	4 (3.7%)
Cardiac arrhythmia	4 (3.7%)
Coronary artery bypass surgery	2 (1.9%)
Atrial fibrillation	5 (4.6%)
Surgical approach:	
Direct lateral	61 (56.5%)
Direct anterior	47 (43.5)
Mobility aid use:	
None	83 (76.9%)
Single Point Cane	20 (18.5%)
Rollator Walker	5 (4.6%)
Among mobility aid users:	
Intermittent use	17 (15.7%)
All the time	8 (7.4%)
Use mobility aid because of total hip arthroplasty	10 (9.3%)
Self-report of physical activity level:	
Sedentary	19 (17.6%)
Moderate	45 (41.7%)
Vigorous	44 (40.7%)
Montreal Cognitive Assessment	26.2 ± 2.7 [18-30]
Western Ontario McMaster Universities Arthritis Index	84.8 ± 14.7 [42.1–100]
Harris Hip Score	92.6 ± 10.6 [58–100]
SF-12 Mental sub-score	55.1 ± 8.5 [29–65.1]
SF-12 Physical sub-score	45.3 ± 101 [18–58.9]

### Falls questionnaire

The importance of falling compared to other health concerns was of a moderate to high importance (6.8 ± 2.9). The majority of participants (*n* = 98, 90.7%) believed falls can be prevented after THA, yet 13 (12.0%) participants indicated they thought they would fall in the next 12 months. Only 47 (43.5%) people indicated being taught how to prevent falls during their post-surgery THA rehabilitation.

**Fig. 1:**
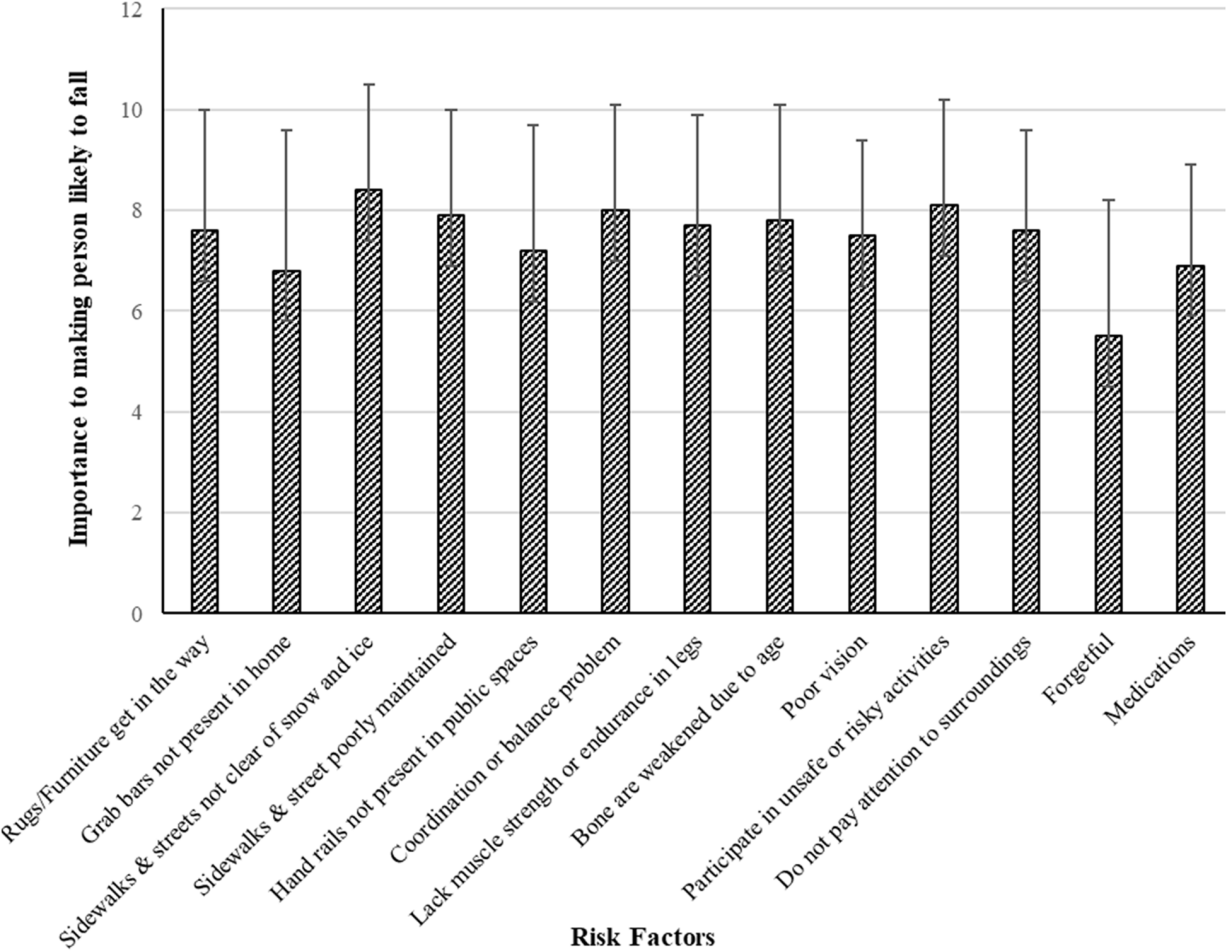
Knowledge of risk factors for falls among older adults who were 12 months after a unilateral total hip arthroplasty for osteoarthritis. (*n* = 108)

In the knowledge of falls risk factors, average scores were greater than 7.0 for the majority of risk factors indicating people believed there was a moderate likelihood to increase the risk of falling. Lower average scores were found for “people are likely to fall because they are forgetful” at 5.5 ± 2.7 and “people are likely to fall because they take many medications” at 6.9 ± 2.0. (Fig. 1) The scores on each risk factor ranged from 0 to 10, indicating a wide variation in perceived importance from low to high likelihood assigned to the risk factors.

A total of 101 modifications were completed by 67 participants to reduce the risk of falling after THA. The types of modifications implemented among the sample were categorized as: nothing at 41.4%, physical environment at 37.4%, behavioral changes at 17.2%, exercise at 2% and support while mobilizing and approach to movement both at 1%.

### Balance confidence

Total scores on the ABC scale ranged from 30.6% to 100.0% with an average score of 84.4 ± 15.5% indicating high balance confidence with activities. Confidence was highest for question 4 (“reach for a small can off a shelf at eye level”) at 96.7 ± 9.0% and lowest for questions 16 (“walk outside on icy sidewalks”) at 57.2 ± 30.7%.

### Physical performance measures

The 6-m Walk Test yielded an average velocity of 1.09 ± 0.22 m/sec and values ranged from 0.35 m/s to 1.57 m/s. Thirty-four (31.5%) people had values below 1 m/s. On the 30CST, the average score was 10.4 ± 3.8 repetitions (range of 0 to 21) and when compared to normative data stratified by age, 30–100% of the age brackets were below age-based values. (Supplementary Table 1) The TUG score was 10.78 ± 3.21 s with values ranging from 6.47 to 32.38 s. Between 63.2% and 83.7% of the age-stratified sample did not achieve age-referenced normative values. (Supplementary Table 2)

### Falls Risk for Older People—Community Setting Questionnaire (FROP-Com)

Scores ranged from 2 – 20 with an average of 8.2 ± 3.6 indicating an overall mild falls risk. A total of nineteen risk factors were identified using the FROP-Com, with an average of 5.4 ± 2.0 risk factors for the sample. The most prevalent risk factors were: medical condition affecting balance/mobility (100.0%), number of prescribed medications (88.0%) and number of alcoholic drinks in the past week (62.0%).

## Discussion

The study found 23.2% of participants had at least one fall in the 12 months after the THA. Just over half of the sample implemented falls prevention strategies after the surgery with the greatest focus on changes made to the physical environment, such as installing grab bars. Importantly, only 43.7% of participants remembered being taught about falls prevention during their rehabilitation after the THA. Yet, knowledge of falls risk factors demonstrated the sample perceived known risks to have a moderate likelihood of leading to falls and there was high balance confidence. Physical measures of lower extremity strength, gait and functional mobility were reduced compared to normative values of community-dwelling older adults for the majority of the participants. These areas of deficits are associated with an increased risk of falls [36]. Using the FROP-Com the majority of participants were identified at a mild risk of future falls, though the evaluation of balance and gait deficits have a limited number of questions in the scale and assessment is through observation only rather than performance-based outcome measures. Therefore, we suggest the FROP-Com should not be used in isolation for identifying and stratifying falls risk in this relatively high functioning patient population. Use of FROP-Com in conjunction with physical-performance tests to identify deficits commonly found after THA is suggested to facilitate interventions for modifiable factors of strength, balance, and gait.

Research has found that older adults at an increased risk for falls do not receive routine systematic falls risk assessment during rehabilitation [33]. In our sample, less than half of the participants remembered receiving falls prevention information during their rehabilitation after THA. Current falls prevention guidelines focus on tertiary prevention, the assessment and implementation of prevention strategies in response to people falling, while the role of secondary prevention (risk factors are identifiable in the absence of a falls history) has a less prominent role [16]. In our sample, the THA was an elective procedure for osteoarthritis and not performed in response to a fall-related injury, unlike the surgical management of a hip fracture. Therefore, the impetus to include falls prevention after THA for osteoarthritis was likely reduced among health care professionals as there is no index event driving future prevention. Future research is needed to understand the facilitators and barriers for physiotherapists to include falls prevention as part of routine THA rehabilitation.

Our sample of people rated falls as a moderate to high health topic compared to their other health conditions, understood the importance of falls risk factors and believed that falls were preventable. These findings are consistent with the work by Braun et al. [19] on community-dwelling older adults. Yet there was a large range of scores for the 13 targeted falls risk factors evaluated. The fact that answers ranged from 0 to 10 for the majority of risk factors indicated people after THA need an enhanced understanding of falls to better self-evaluate and implement meaningful prevention strategies.

Our study found that the most common strategy used by the participants was environmental modifications, such as installing grab bars and removing loose rugs or clutter. This finding is consistent with research that has evaluated falls prevention strategy implementation after prosthetic rehabilitation for people with a lower extremity amputation [35] and older adults discharged from acute and rehabilitation hospitals [20]. The method of delivery and content of falls prevention information is very important to the uptake and application of strategies by older adults [34]. Research to evaluate the content of falls education programs after THA is also suggested. Existing research has also demonstrated older adults implement a limited number of falls prevention strategies which could reflect limited knowledge of available options [20] or a focus on strategies that can receive supported funding such as equipment needs that are prescribed by healthcare professionals [35]. Few people reported exercise as an intervention and in light of the large percentage of the sample with deficits in balance, gait and lower extremity strength at 12 months after THA this is an area of great importance to advocate as a falls prevention strategy.

Brander et al. [17] proposed that falls risk assessment should be performed starting at 3–6 months after THA, a time frame consistent with the occurrence of 80.6% of falls in our study. Yet, the occurrence of falls in our sample was lower than that previously reported among people 12 months after THA [11]. The study by Ikutomo et al. [11] included only women and it is well established that women have a higher falls risk and occurrence of falls than males [36]. Using clinical tests that targeted predicted areas of physical function limitation after THA, our study found deficits in gait, lower extremity strength and functional mobility were very prevalent, findings that are consistent with the existing literature in this patient population [6, 7]. Assessment of THA-specific deficits, in conjunction with a comprehensive evaluation of global falls risk factors for the community-dwelling older adult would be suggested for identification and initiation of prevention strategies.

### Limitations

The main limitation of our study is the cross-sectional design as this lead to under-reporting of falls in the previous year, though retrospective reporting over a 12 month time frame is supported as valid [37]. Therefore our results likely represent a conservative estimate of the true falls prevalence. Strengths of the study include an evaluation of the participants’ understanding of falls risk factors and falls prevention strategies allowing insight for the development of falls education programs. While the study design was cross-sectional, a single assessment to determine falls risk is consistent with clinical practice. In the absence of test score thresholds to identify future falls risk for the physical performance tests used in this study, the comparison of physical function against normative data is an acceptable means to determine benchmarks for abilities in the population. Additionally, we utilized a valid measure of falls risk for older adults and standardized clinical tests that targeted areas where deficits would likely be present after THA surgery.

## Conclusions

Older adults one-year after a THA rated falls as a moderately important health topic compared to their other health conditions, falls risk factors were understood to be important and most believed that falls were preventable. Yet, there was a large variation in specific knowledge for factors that increase falls risk. Less than half of the sample reported receiving falls prevention education. Most falls prevention strategies implemented environmental modifications with exercise being the least reported strategy. Using performance-based clinical tests, most of the sample had deficits in gait, lower extremity strength and functional mobility that would be associated with an increased falls risk. People after THA are at an increased risk of falls due to functional deficits but have limited exposure to falls prevention education and implement a limited range of prevention strategies.

## Supplementary Information

**Additional file 1:** **Supplementary Table 1**. 30-Second Chair Stand Test results in people 12 months after total hip arthroplasty compared to normative values stratified by age and sex. (*n*=108). **Supplementary Table 2. **Timed Up-and-Go Test scores in people 12 months after total hip arthroplasty compared to normative values stratified by age. (*n*=107). 

## Data Availability

The datasets generated and/or analyzed during the current study are not publicly available but may be available from the corresponding author on reasonable request.

## Abbreviations

6mWT: 6-Meters Walk Test
30CST: 30-Second Chair Stand Test
ABC: Activities-specific Balance Confidence Scale
FROP-Com: Falls Risk for Older People- Community Setting questionnaire
OA: Osteoarthritis
SF-12: Short-Form 12
THA: Total Hip Arthroplasty
TUG: Timed-up and Go Test
WOMAC: Western Ontario and McMaster Universities Arthritis Index
