# Supplementary material for: Implementation of falls risk evaluation at one-year after total hip arthroplasty: a cross-sectional study
Source: Arch Physiother. 2022 Jul 15;12:16. doi: 10.1186/s40945-022-00141-6 (PMC9284763; doi:10.1186/s40945-022-00141-6)
Supplement: Supplementary file 1 — Additional file 1: Supplementary Table 1. 30-Second Chair Stand Test results in people 12 months after total hip arthroplasty compared to normative values stratified by age and sex. (n=108). Supplementary Table 2. Timed Up-and-Go Test scores in people 12 months after total hip arthroplasty compared to normative values stratified by age. (n=107). [file 40945_2022_141_MOESM1_ESM.docx]

**Supplementary Tables**

**Supplementary Table 1**. 30-Second Chair Stand Test results in people 12 months after total hip arthroplasty compared to normative values stratified by age and sex. (n=108)

| **Age Group (Male)** | **Number of participants** | **Mean ± SD**  **(Repetitions)** | **Normative Values**(Rikli & Jones, 1999)  **(Repetitions)** | **Participants below normative values, n (%)** |
| --- | --- | --- | --- | --- |
| 60 – 64 | 5 | 10.8 ± 2.2 | 14 | 4 (80.0) |
| 65 – 69 | 9 | 12.0 ± 3.1 | 12 | 7 (77.8) |
| 70 – 74 | 13 | 9.9 ± 4.3 | 12 | 9 (69.2) |
| 75 – 79 | 9 | 10.6 ± 2.6 | 10 | 4 (44.4) |
| 80 – 84 | 6 | 11.3 ± 2.9 | 10 | 3 (50.0) |
| 85 – 89 | 1 | 14 | 8 | 0 (0.0) |
| **Age Group (Female)** | **Number of participants** | **Mean ± SD**  **(Repetitions)** | **Normative Value**(Rikli & Jones, 1999)  **(Repetitions)** | **Participants below normative values, n (%)** |
| 60 – 64 | 8 | 11.8 ± 2.6 | 12 | 5 (62.5) |
| 65 – 69 | 21 | 11.1 ± 3.1 | 11 | 15 (71.4) |
| 70 – 74 | 13 | 10.9 ± 1.9 | 10 | 5 (38.5) |
| 75 – 79 | 11 | 8.9 ± 6.1 | 10 | 7 (63.6) |
| 80 – 84 | 11 | 6.6 ± 4.0 | 9 | 8 (72.7) |
| 85 – 89 | 1 | 8 | 8 | 1 (100.0) |

**Supplementary Table 2.** Timed Up-and-Go Test scores in people 12 months after total hip arthroplasty compared to normative values stratified by age. (n=107)

| **Age Group** | **Number of participants** | **Mean ± SD**  **(Seconds)** | **Normative Data** (Steffen et al., 2002) **(Seconds)** | **Participants below normative values, n (%)** |
| --- | --- | --- | --- | --- |
| 60 – 69 | 43 | 9.84 ± 2.47 | 8 | 36 (83.7) |
| 70 – 79 | 45 | 10.70 ± 2.13 | 9 | 35 (77.8) |
| 80 – 89 | 19 | 13.11 ± 5.26 | 10 | 12 (63.2) |
